# Do geography and institutions affect entrepreneurs’ future business plans? Insights from Greece

**DOI:** 10.1186/s13731-023-00266-3

**Published:** 2023-02-24

**Authors:** Evangelos Rasvanis, Vassilis Tselios

**Affiliations:** 1 Department of Planning and Regional Development, University of Thessaly, Pedion Areos, 383 34 Volos, Greece; 2 Department of Economic and Regional Development, Panteion University of Social and Political Sciences, 176 71 Athens, Greece

**Keywords:** Geography, Institutions, Business expansion, Business selling, Greece, D02, M20, P28, R12

## Abstract

It is widely known that both geography and institutions determine the location of an investment. However, little is known about the impact of these factors on entrepreneurs’ future business plans. Using a questionnaire survey, this paper examines whether the 'first-' and 'second-nature' geography and institutions affect the plans of entrepreneurs to expand or sell their businesses in Greece. The analysis shows that entrepreneurs intend to expand their businesses when the importance of the country's relative geographical location to the international market increases, but that of localisation economies decreases. There is also evidence that entrepreneurs aim to sell their businesses to domestic investors when sea access, the natural environment, the agglomeration economies, the location of Greece and the local governance are ideal for the business performance in the country.

**Supplementary Information:**

The online version contains supplementary material available at 10.1186/s13731-023-00266-3.

## Introduction

Geography matters for entrepreneurs’ location choices. The natural geographical advantages of an area, known as ‘first-nature’ of geography factors (Krugman, 1993), can play an important role in attracting economic activities, especially those that are directly dependent on the natural endowments. For example, the wind turbines of renewable energy investments are located in windy areas and the sea-tourism investments in coastal areas. Although many scholars have examined the ‘first-nature’ geography as a locational determinant of investments, and especially of foreign direct investments (Lu et al., 2020; Rasvanis & Tselios, 2022), little is known about its impact on entrepreneurs’ future business plans. The geographical proximity between businesses and/or people, known as agglomeration economies or ‘second-nature’ geography, is a key locational determinant of investments (McCann & Shefer, 2003). There is some evidence that ‘second-nature’ geography can be an important factor for the expansion of businesses (Fotopoulos & Louri, 2000; Yüzer & Yüzer, 2014). For example, many businesses are more likely to survive and grow in metropolitan areas, which are full of economies of scale, than in other areas (Fotopoulos & Louri, 2000), because metropolitan areas facilitate the development of businesses, through the direct diffusion of knowledge, the large supply of higher skilled labour, and access to the transportation infrastructure (Yüzer & Yüzer, 2014). It is noteworthy that although digital transformations have helped to drastically reduce the transport and communication costs, agglomeration effects remain vital for businesses (Glaeser, 2010).

Institutions matter for entrepreneurs’ location choices as well. The high quality of institutions can be a key factor in attracting investments. For example, the high quality of local governance can help strengthen the competitiveness of an area, through the equal treatment and support of businesses operating in that area (Cheng & Yiu, 2016). Greater emphasis on shaping investors' development aspirations has been put on factors related to finance accessibility tools, such as public credit guarantee schemes (Martín-García & Morán Santor, 2021), to the availability of external finance (Becchetti & Trovato, 2002), to the trade environment (Gupta et al., 2013), to tax policy (Kassa, 2021) and to infrastructure (Kumar, 2007), and less on the role of institutional aspects, such as corruption and local governance, when it comes to entrepreneurs’ future business plans (e.g. Karlsson & Acs, 2002; Saeedikiya et al., 2021).

This paper aims to fill the significant gap in the literature about the role of geography and institutions in entrepreneurs’ future business plans, by examining the case of Greece, a small European Union (EU) country with rich natural endowments, but with a high agglomeration of economic activities and relatively low quality of institutions. The overarching aim of this paper is that the findings can serve as a guide for policy analysts on the decisions they could take to boost entrepreneurship in Greece and thus the competitiveness of the Greek economy.

Greece is an interesting case-study for many reasons. First, it is a country in the so-called ‘European South’, whose production model in recent decades is overwhelmingly based on services aimed at domestic consumption. The manufacturing sector is limited, and except for the pharmaceutical and chemical industries, it consists of low-skilled industries. Most businesses in all sectors are very small, such as family businesses, employing up to ten employees. The technology they use is imported and innovation is generally absent, apart from some individual efforts by people and businesses. This model is not in line with the fourth industrial revolution and the digital transformation of the European and world economy. After the global financial crisis of 2008, in which the Greek economy experienced the largest recession among the EU countries, policy makers are keen to reform its economy and move the country forward.

Despite its outdated productive model, Greece, as a whole, has some indisputable natural geographical advantages. The access afforded to almost all of its regions to the sea, the very good climatic conditions, the existence of fertile land in many of its regions and the possibility of using renewable energy sources are some of the country’s advantages. The country's economy relies heavily on these natural advantages and it comes as no surprise that tourism is the only ‘heavy industry’ in the country (Samitas et al., 2018). Greece needs to maintain, expand, and attract new investments in its territory, in order to combat its serious public debt problems and to develop its economy.

As for the concentration of economic activities, they are observed mainly in the two major metropolitan regions of the country: the Region of Attica, which is home to the capital of Athens; and the Region of Central Macedonia, which is home to Thessaloniki. These two regions are also heavily populated. In fact, their population in total exceeds 50% of the total population of the country. In the case of the Attica Region, the accumulation of economic activities extends beyond its regional limits (Petrakos & Psycharis, 2016).

Although Greece is considered to be the cradle of democracy, the functioning of institutions in the country is not ideal. According to the Corruption Perceptions Index 2020, the country ranked 59th among 180 countries worldwide, slightly better than only 5 countries belonging to the group of former Transition Economies of the 27 EU member countries (Transparency International, 2021). Moreover, the public administration in the country is not good, as 76% of the Greek citizens believe that the provision of public services is poor, which is the worst performance among the member-states of the EU (Directorate-General for Communication, 2021). The role of local governments in Greece is limited compared to Central and Northern European countries, as it largely depends on the funding of the central government.

The remainder of this paper is structured as follows. The next section reviews the existing literature related to the geographical and institutional factors that may affect investors’ plans. "Methodology" section describes the data, the variables and the research method, and presents the descriptive statistics. "Logistic regressions results" and "Discussion" sections present and discuss the results obtained from the logistic regression models, respectively. The final section concludes with implications about the policies that could contribute to business retention and expansion.

## Literature review

### Geography-related locational factors

There are many geography-related characteristics which influence the location of investments and, thus, the distribution of economic activities. Traditionally, these characteristics are distinguished between two groups: the first group is related to the physical landscape and natural geographical circumstances (i.e. ‘first-nature’ of geography factors), such as sea access, the presence of natural resources and the quality of the natural environment; and the second group is related to agglomeration economies (i.e. ‘second-nature’ of geography factors), such as the proximity to competitors, the location of the country in relation to the international market and the proximity to a capital. But, do these geography-related locational factors affect the business performance?

First of all*,* the low cost of maritime transport is a strong advantage for businesses that are located or intend to settle in coastal areas and in areas near navigable rivers or major lakes, because it can help them to expand their production and to better serve foreign markets (DÉMurger et al., 2002; Henderson et al., 2017). Sea access has been a very important factor in the development of trade since ancient times (such as Mesopotamia, ancient Egypt and ancient Greece), as areas with access to the sea were pioneers. Gallup et al. (1999) noticed that landlocked countries may be at a particular disadvantage, because cross-border labour migration is harder than internal migration and additional costs may be imposed on them by coastal countries. Finally, there are studies (e.g. Coughlin & Segev, 2000; Lu et al., 2020) that argue that maritime access is a key determinant in attracting foreign investors.

The presence of natural resources in an area can be a location advantage for a business. The proximity to natural resources can be a determining factor for the development of a business, because it can easily obtain access to raw materials either without or with low transportation costs. Ellison and Glaeser (1999) found that the location of industries in the United States is affected by the existence of natural resources, while Wiggins and Proctor (2001) support the economic role of rural areas based on the fact that natural resources are located in specific places and cannot be moved.

The quality of the natural environment is evaluated as an important location factor of a business (e.g. high-tech industries) for executives and high-skilled workers, but as a less important factor for the establishment or expansion of manufacturing companies. Malecki and Bradbury’s (1992) research shows that one of the top five location characteristics for both R&D facilities and their employees is environmental quality. Although this research shows that employees and their employers are more satisfied in urban agglomerations, factors such as environmental quality, cost of housing, recreational opportunities and climate are the most important characteristics in evaluating an ideal future location (Malecki & Bradbury, 1992). Leigh and Blakely (2016) argue that the location of businesses no longer depends on proximity to natural resources (i.e. raw materials), because technology and digital transformations have certainly reduced the importance of distance. Instead, businesses are usually more interested in a location where natural and social factors work together to create a good quality of life and a business environment which will be economically viable. This will attract and retain highly-educated and skilled workers, who seek out areas that have less gas pollution, more green spaces and pleasant living conditions (Boon, 2003; Konsolas, 1997).

As for the factors related to the interaction between economic agents (‘second-nature’ of geography factors), they significantly determine the location of economic activities. According to the New Economic Geography theory (Krugman, 1998), these factors describe the advantages of agglomeration economies, which are endogenous and independent of the natural endowments (Schmutzler, 1999). There are two types of agglomeration economies (also known as agglomeration externalities or effects): the localisation economies and the urbanisation economies. The localisation economies refer to the benefits which derive from being located close to other firms in the same or a similar industry. These economies exist mainly due to the job specialisation, the lowest prices that can be achieved in input supplies, the provision of specialised support services, and the potential dissemination of knowledge. The localisation economies can be proxied by the proximity to competitors (Rasvanis & Tselios, 2021). The urbanisation economies refer to the benefits which derive from being located in large urban centres. Some of these benefits are proximity to the market, labour market pooling and the promotion of innovation and productivity growth, due to the knowledge transfer among different industries. There are only a few studies that have examined the role of agglomeration externalities in the performance and survival of businesses, and especially of new businesses (Falck, 2007; Neffke et al., 2012). These studies have found mixed results, as the agglomeration economies can either benefit a business or have detrimental effects on business survival. As the largest urbanisation economies are observed in the capitals of most European countries (e.g. Paris, London and Athens), the urbanisation economies can be proxied by the proximity to the capital (Louri, 1988; Rasvanis & Tselios, 2021).

Finally, the geographical location of the country on the world map, which is related to factors such as proximity and relative distance from other economically and commercially developed countries or emerging markets (‘second-nature’ geography), may play a central role in both attracting and developing businesses in this country.^1^ For example, Moreno and Trehan (1997) found that distance is an important determinant of trade and that countries close to large markets tend to invest a high percentage of their output. Thus, countries that have a central position and easy access to large international markets have a comparative advantage over countries that do not have this geographical advantage. This is the case of Greece, as its geographical location—at the south-eastern tip of Europe and at the maritime crossroads of Europe, Asia, and Africa—facilitates maritime communication with the large Asian market. Moreover, Coşar and Fajgelbaum (2016: p. 25) state that ‘the geographical advantage of international gates acts as an agglomeration force’.

### Institutions-related locational factors

Although there is no single commonly accepted definition of what institutions are, the importance of their quality is considered crucial both for the economic development of an area (Acemoglu et al., 2002, 2005) and for the entrepreneurship and business expectations for further growth (Kaplan & Pathania, 2010; Nguyen et al., 2013). The quality of institutions mainly involves the functioning of democracy, justice (law enforcement), the protection of property rights, and the absence of corruption. Institutional deficits can act as a deterrent in attracting investment, because they can act as an additional cost to potential investors, and can increase the degree of uncertainty about the future of the return on investment (Daude & Stein, 2007).

One aspect of formal institutions which is based on the imperfect functioning of democracy and law enforcement and is considered very important in the decision-making process of domestic and foreign investors is that of corruption. Corruption could exist due to: the large public sector when corrupt politicians increase the rents from illegal behaviour; the complexity of legislation and the lack of clear rules in the operation of the market, as the rule of law is crucial for democracy and social justice; the lack of economic competition, government failures and ineffective price controls; and the lack of cultural and human values, among others (Lambsdorff, 2006). Existing empirical studies on businesses’ survival and growth indicate mixed results. Some of them support the claim that corruption has a detrimental effect on the performance and survival of domestic businesses (e.g. Nam et al., 2020), while other studies suggest that bribery (which is a proxy for corruption) facilitates innovation and in some cases firm growth, allowing companies to bypass regulatory barriers (Ayaydın & Hayaloglu, 2014; Karaman Kabadurmus & Sylwester, 2020).

Another institutional aspect is the functioning and practices of local government. Local governments, depending on their degree of autonomy from the central government, can help local businesses grow. This can be done either through the financing of local governments to local businesses, the contribution of local governments to the promotion of the products and services of local businesses in other markets, inside or outside the country, or even through the provision of incentives and tax relief. The importance of the local government in businesses’ performance is more profound in the developed countries than in the less-developed countries, because local government can shape the educational attainment and the promotion of research and innovation, and, thus, can contribute to the increase of highly-trained and specialised jobs. This can help local companies to apply innovative methods of production and to promote innovative products. Finally, the support and contribution to the development of local businesses by the local government entails increased tax and other revenues, which are likely to be borne by the local community (Olsson et al., 2020).

## Methodology

### Data

In order to investigate the impact of geographical and institutional factors on the future business plans of the entrepreneurs located in Greece, after controlling for some business and location characteristics, we have relied on the opinions expressed by the managers and/or owners of the domestic and foreign-owned businesses operating in the country. We have used data derived from a self-administered questionnaire survey. The reference population of the survey was the secondary and tertiary sectors of the Greek economy However, the focus of this survey is on the sub-sectors of tourism, and transport and logistics. Tourism is undeniably the most important economic activity of the country. According to the data of the Hellenic Statistical Authority for 2018, the number of legal units operating in the industry of tourism amounts to 15% of all legal units in the tertiary sector, while its total contribution (direct and indirect) to Greece's GDP ranged between 25.7 and 30.9% (INSETE, 2019). Transport and logistics is an ever-growing industry in the country. Greece is becoming an important logistics center for the rest of Europe, exploiting mainly the large ports of Piraeus and Thessaloniki. In this survey, we excluded the primary-sector investments, because the vast majority of them consist of very-small, individual or family businesses, whose owners are difficult to identify as potential respondents to a questionnaire survey.We used the stratified sampling method, with the following four strata: (a) tourism businesses (accommodation) of any legal form, (b) Public Limited Companies (PLCs), Private Limited Companies (Ltds) and Limited Liability Companies (LLCs) of the transport and logistics sector, (c) manufacturing PLCs, Ltds and LLCs, and (d) PLCs, Ltds and LLCs of the other sub-sectors of the services sector (tertiary sector). The population, the sample per stratum and the composition of the survey respondents are presented, in more detail, in Table 1.

**Table 1:** Sampling frame, sample and survey respondents

Stratum	Industry	Sampling Frame	Sampling (Confirmed questionnaires received)	Survey Respondents	Percentage (%) in total participants
1st	Tourism (Accommodation)	3800	2000	431	42
2nd	Transport and Logistics	988	901	193	19
3rd	Manufacturing sector • Manufacture of food and beverage and tobacco products • Manufacture of pharmaceutical products and pharmaceutical preparations • Manufacture of soap and detergents • Manufacture of rubber and plastic products • Manufacture of chemicals and chemical products • Manufacture of textile, wearing apparel and leather products • Manufacture of paper and paper products • Manufacture of fabricated metal products • Construction • Energy (Electric power generation, transmission and distribution and manufacture of gas)	520	950	223	22
4th	Services sector except for tourism, transport and logistics • Wholesale and retail trade of food, beverages and tobacco • Computer programming, consultancy and related activities • ICT and Information service activities • Telecommunications • Architectural and engineering activities; technical testing and analysis • Real estate activities • Human health activities • Other business activities	9959	1350	168	17

Authors’ elaboration. Sampling frame data are based on www.findbiz.gr (ICAP) database (2018)

The collection of responses started in February 2018, was interrupted in December 2018 and continued with the addition of new data in the first quarter of 2020, until the suspension of most economic activities in Greece at the end of March 2020 due to the Covid-19 pandemic. The business data and contact details were extracted from the findbiz/ICAP database (ICAP CRIF, 2018). The specialised application 'eval and go' was used to deliver the questionnaires to the business owners and/or managers. Nevertheless several telephone contacts followed the sending out of the questionnaires, in order to convince respondents to take part in the survey. The overall response rate was around 19.5%, after removing responses from managers and/or owners of businesses who either did not answer many questions or they answered in an unusually short time.^2^ Hence, the final database consists of 1015 respondents, 78% of which are businesspeople in the services sector and 22% businesspeople in the manufacturing sector. These participant rates approximate the data of the real Greek economy, as the gross value added (GVA) of the services sector represents 80 per cent, while the GVA of the manufacturing sector is slightly below 20% (Eurostat, 2021).

### Econometric specification

The questionnaire survey^3^ covers two questions regarding business expansion and business selling. The first question is about an investor’s intention to further expand his/her business in the Greek territory. Τhe second one asks whether an investor wants to sell his/her business and, if so, to whom (i.e. to an investor of what origin). The second question could show us not only an investor's intention to sell his/her business, but also the origin of the future investor. Hence, respondents had to choose between multiple answers, i.e. whether or not they intend to sell their businesses, and if they intend to do so, what would be the origin of the proposed acquirer.

The likelihood of the expansion of a business or the likelihood of the selling of a business to a domestic or foreign investor is given by the following logistic regression.

1 $${y}_{i}={a}_{0}+{FirstNature}_{\iota }{\beta }_{1}+{SecondNature}_{\iota }{\beta }_{2}+{Institutions}_{\iota }{\beta }_{3}+{Controls}_{\iota }{\beta }_{4}+{\varepsilon }_{\iota }$$

where in the case of the probability of the expansion of a business in the Greek territory, ${y}_{i}$ is a dichotomous variable divided into two categories: (a) investor (or manager) wants to expand his/her business, and (b) investor (or manager) does not want to expand his/her business (reference category)^4^; while in the case of the probability of the business being sold, ${y}_{i}$ is a categorical variable divided into the four categories: (a) maybe, (b) it will be sold to a domestic investor, (c) it will be sold to a foreign investor, and (d) no, the investment is not for sale (reference category). ${FirstNature}_{\iota }$ and ${SecondNature}_{\iota }$ are vectors of the geography-related locational factors of business *i*, ${Institutions}_{\iota }$ is a vector of the institutions-related locational factors of business *i,* and ${Controls}_{\iota }$ is a vector of the control characteristics of business *i*. ${a}_{0}$ is a constant, ${\beta }_{1}, {\beta }_{2}, {\beta }_{3} and$ ${\beta }_{4}$ are vector coefficients, and ${\varepsilon }_{\iota }$ represents the disturbance term.

By developing Eq. 1, we end up with the following specification:

2 $$\begin{aligned}logit\left({P}_{i}\right)&= \mathrm{ln}\left(\frac{{P}_{i}}{{P}_{j}}\right)= {a}_{0}+{\gamma }_{1}{SeaAccess}_{i}\\ & \quad+ {\gamma }_{2}{NaturalResources}_{i}+{\gamma }_{3}{NaturalEnvironment}_{i} \\ & \quad+{\gamma }_{4}{ProximityCompetitors}_{i}+{\gamma }_{5}{ProximityCapital}_{i}\\ & \quad+{\gamma }_{6}{CountryLocation}_{i}+{\gamma }_{7}{Corruption}_{i}\\ & \quad+{\gamma }_{8}{LocalGovernance}_{i}+{\gamma }_{9}{TaxPolicy}_{i}\\ & \quad+{\gamma }_{10}{Infrastructure}_{i}+{\gamma }_{11}{Origin}_{i}\\ & \quad+{\gamma }_{12}{Sector}_{i}+{\gamma }_{13}{Size}_{i}\\ & \quad+{\gamma }_{14}{Establishments}_{i}+{\varepsilon }_{\iota }\end{aligned}$$

where $logit\left({P}_{i}\right)$ is the natural logarithm (*ln*) of the $\frac{{P}_{i}}{{P}_{j}}$ odds, i.e. $\mathrm{ln}\left(\frac{{P}_{i}}{{P}_{j}}\right)$. In the case of business expansion, ${P}_{i}$ is the probability of a business being expanded in the Greek territory and ${P}_{j}$ is the probability of a business not being expanded, while in the case of the business being sold, ${P}_{i}$ presents the odds of the probability of an entrepreneur who is not sure whether he/she aims to sell his/her business or the probability of an entrepreneur who will definitely sell it either to a domestic investor or to a foreign investor, and ${P}_{j}$ is the probability of a business not being sold.

The variables used to measure the impact of the natural endowments of a specific location or the country as a whole (‘first-nature’ geography) on an investor’s future plans are his/her views about the significance of the proximity to the sea ($$SeaAccess)$$, the access to natural resources ($$NaturalResources$$) and the quality of the natural environment ($$NaturalEnvironment)$$. To analyse the role of proximity between economic agents (‘second-nature’ geography) in an investor’s intentions about his/her business, we define three variables: (a) the proximity to the firms of the same or a similar industry $$(ProximityCompetitors)$$, which is a proxy for localisation externalities, (b) the proximity to the Greek capital ($$ProximityCapital)$$, which is a proxy for urbanisation externalities, because the Greek capital –i.e. Athens– is the largest urban centre, where most economic activities are concentrated, and (c) the location of the country ($$CountryLocation$$), which expresses the relative location of Greece on the globe. To measure the impact of institutions on the investors’ future plans, we rely on two variables: (a) the corruption of the country ($$Corruption$$), and (b) the local governance of the country ($$LocalGovernance$$). All these variables are measured by the responses of the survey participants on graded scale questions, and in particular on a 7-point Likert scale. We preferred this scale than the 5-point Likert scale, because the 7-point scale offers more options to the respondents and captures better the opinion of the respondent. Hence, it provides better accuracy in the results.In the analysis, we control for the role of two important factors, which are measured by a 7-point Likert scale: (a) the tax policy $$(TaxPolicy)$$ followed by the government of a region or the whole country (Kassa, 2021), and (b) the quality of the transport and communication infrastructure ($$Infrastructure)$$ (Audretsch et al., 2015). First, tax policy is a key factor both in attracting new investment and in the viability of those already established in a country. The reduction of the tax on business income favours an increase in investments (e.g. Kitao, 2008). The study of Da Rin et al. (2011) shows the importance of corporate taxation in businesses’ performance located in 17 Western European countries, but the reduction of corporate tax rates is more effective in countries with better quality institutions, which, in turn, could contribute to the entrance of new businesses into the market. The study of Baliamoune-Lutz (2015) shows that tax progressivity has a negative impact on young entrepreneurs located in the Organization for Economic Co-operation and Development (OECD) countries, but there is no evidence about the existing entrepreneurs. Second, investments in the transport and telecommunications infrastructure, which are large-scale investments, significantly affect both individuals and businesses. These investments are usually public or in some cases implemented through public–private partnerships (Audretsch et al., 2015). Kaur et al. (2016) point out that better transport services led not only to a reduction in the transport cost within a country, but also, to a reduction in the cost of imports and exports. However, the impact of infrastructure on businesses, and especially on start-ups, varies, depending on the type of infrastructure. For example, broadband and rail infrastructure have a much more significant positive impact on start-ups compared to highways (Audretsch et al., 2015).

Apart from the above two locational factors, we also control for the impact of the characteristics of the businesses. More specifically, we control for the origin of business *i* (${Origin}_{i}$), through a dummy variable, which takes the value 0 when the business is domestic, and the value 1 when the business is foreign-owned; the sector in which business *i* is classified (${Sector}_{i}$), through a dummy variable, which takes the value 0 when the business belongs to the manufacturing or the energy sector and the value 1 when it belongs to the services sector; and the size of business *i* (${Size}_{i}$), which is a variable based on the number of employees and is divided into the following four categories: (a) very small (up to 10 employees), (b) small (10 to 49 employees), (c) medium (50 to 249 employees) and (d) large (equal to or greater than 250 employees). Finally, we control for the number of establishments of a business *i* (${Establishments}_{i}$) using a dichotomous variable, which takes the value 0 when there is only one establishment in Greece and the value 1 when there is more than one establishment.

### Descriptive analysis

We first present some descriptive facts for the two dependent variables: business expansion and business selling.

**Fig. 1:**
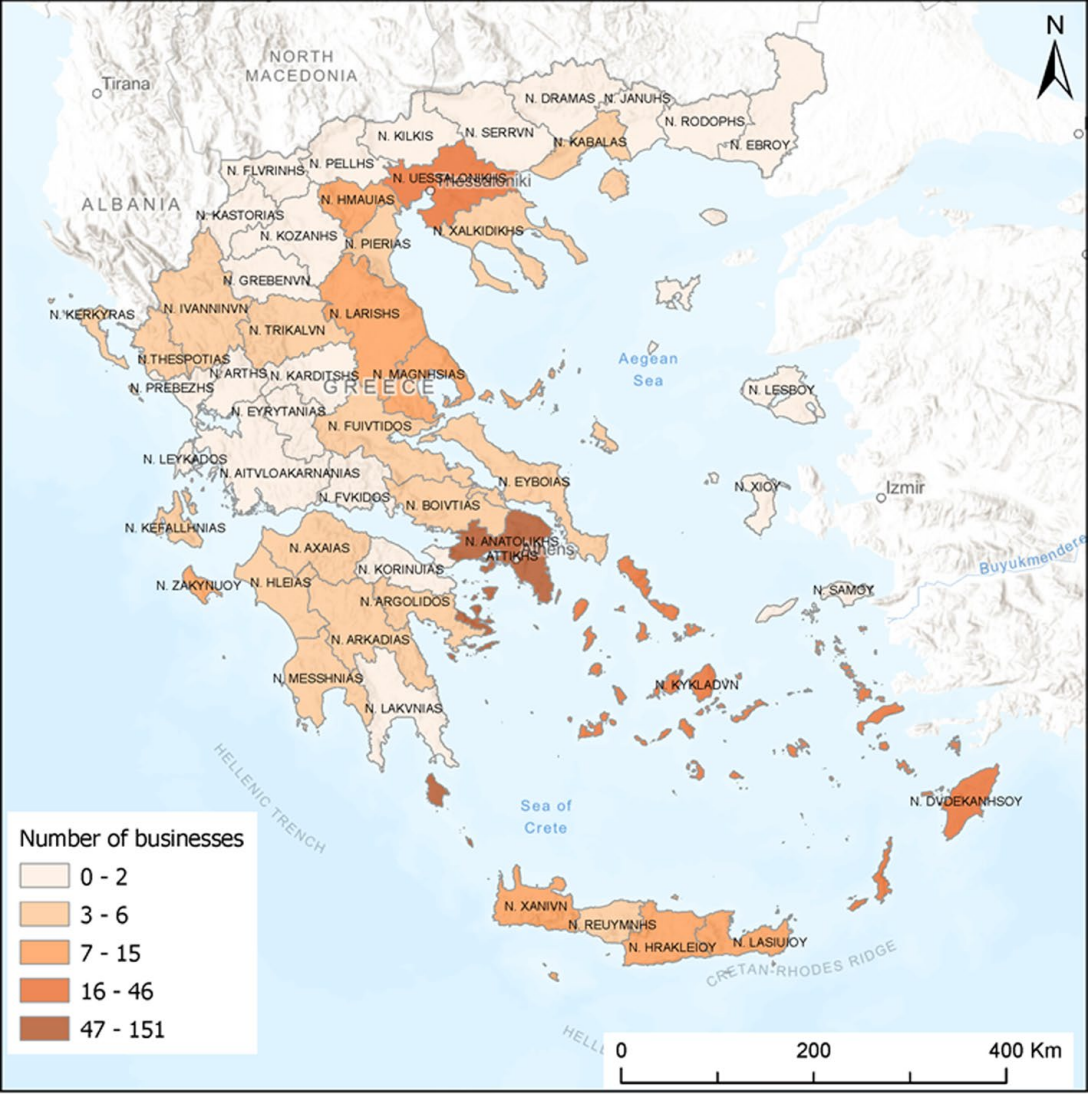
Geographical distribution of business expansion

The majority of entrepreneurs operating in Greece intend to expand their business (402 respondents versus 324 respondents). Figure 1 displays the regional distribution of the business expansion at NUTS III level. We need to clarify that in most businesses (72%), the location of the headquarters of a business coincides with the location of the main production unit of the business. This figure indicates that managers or owners whose businesses are located in the regions with a high concentration of economic activities and with proximity to the sea and large ports (e.g. Attica, Thessaloniki, Magnesia) intend to expand their investments in the Greek territory. These regions have a rather low-quality of natural environment and are the major urban centres of the country (e.g. Athens, Thessaloniki, Heraklion, Larissa).

**Fig. 2:**
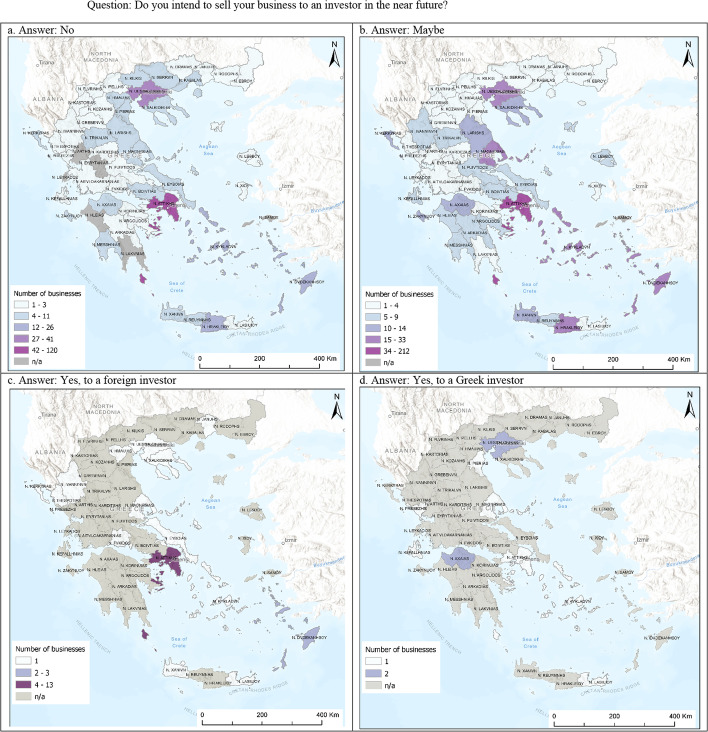
Geographical distribution of business selling. Question: Do you intend to sell your business to an investor in the near future?

Regarding the business selling, 556 entrepreneurs do not have a clear plan to sell their business, 367 do not intend to sell their business in the near future, 40 intend to sell their business, and 52 did not answer. The responses of entrepreneurs are likely to denote the resilience of the Greek businesses. Despite the prolonged economic crisis of 2008, the entrepreneurs have endured and believe that the future will be more auspicious. Most of the respondents who plan to sell their businesses intend to sell them to a foreign investor (29 out of 40), because they probably have an acquisition offer from a foreign rather than a domestic investor. Figure 2 displays the distribution of the business selling at NUTS III level. The businesses that are not planned to be sold to a domestic or foreign investor and those that do not have a clear plan are located in the capital (Athens) or in the second largest city of Greece (Thessaloniki). Apart from the businesses located in Athens or Thessaloniki, those that are located in Magnesia are also likely to be sold in the future. The Prefecture of Attica and the Prefecture of Dodecanese have the most entrepreneurs who intend to sell their businesses to foreign investors, while the Prefectures of Thessaloniki and Achaia, which combine the natural advantage of maritime access with high agglomeration economies, have the most entrepreneurs who intend to sell their businesses to domestic investors.

Table 2 displays the descriptive analysis of all the variables examined. This table shows that the vast majority of businesses (about 70%) located in Greece are either very small or small businesses with up to 49 employees. 66.39% of the businesses have only one establishment. The majority of the Greek businesses are in the service sector. We also observe that tax policy and political stability are rated more highly than the ‘geography’ and ‘institutions’ factors. To stress the importance of tax policy in the performance of a business, it is worth noting that an owner of a company with luxury villas in Peloponnese contacted us and said that the company had changed location because the tax policy and the Greek business environment ‘is counter-productive to viability and growth’.

**Table 2:** Descriptive statistics

Variables	Obs	Mean or Percent	Std. Dev	Min	Max
*‘First-nature’ geography*					
• Sea access	885	4.97	2.16	1	7
• Natural resources	869	3.65	2.10	1	7
• Natural environment	875	4.95	1.63	1	7
*‘Second-nature’ geography*					
• Proximity to competitors	871	4.03	1.88	1	7
• Proximity to the capital	869	3.93	1.98	1	7
• Country location	872	4.10	2.04	1	7
*Institutions*					
• Corruption	821	5.96	1.34	1	7
• Local governance	825	5.53	1.48	1	7
*Controls*					
• Domestic	935	92.12			
• Foreign	80	7.88			
• Manufacturing	223	21.97			
• Services	792	78.03			
• Very small firm	286	28.18			
• Small firm	425	41.87			
• Medium firm	228	22.46			
• Large firm	76	7.49			
• Nr of Establishments = 1	644	66.39			
• Nr of Establishments > 1	326	33.61			
• Tax policy	833	6.54	0.95	1	7
• Infrastructure	817	5.93	1.20	1	7

## Logistic regressions results

### Business expansion

Table 3 presents the binary logistic regression results for the expansion of the existing businesses.^5^ If the statistically significant Odds Ratio (OR) values are higher than 1, the relationship between the predictor and the outcome (i.e. expansion) is positive, while if the statistically significant OR values are lower than 1, this relationship is negative.

**Table 3:** Binary logistic regression results for business expansion

Do you intend to further expand your existing business activity in the Greek territory?		
	Yes	No (base category)
*‘First-nature’ geography*		
Sea access	1.065	
Natural resources	0.927	
Natural environment	0.975	
*‘Second-nature’ geography*		
Proximity to competitors	0.866***	
Proximity to the capital	1.087	
Country location	1.152***	
*Institutions*		
Corruption	1.162	
Local governance	0.975	
*Controls*		
Tax policy	0.902	
Infrastructure	0.874*	
Domestic	Base	
Foreign	0.963	
Manufacturing	Base	
Services	0.841	
Very small firm	Base	
Small firm	0.910	
Medium firm	2.070**	
Large firm	2.104	
Number of Establishments = 1	Base	
Number of Establishments > 1	2.540***	
Constant		1.541
Observations		557
Wald chi^2^		59.42
Prob > chi^2^		0.0000
Pseudo R^2^		0.0899
Log likelihood		− 348.298

^*^p < 0.10; **p < 0.05; ***p < 0.01

The ‘first-nature’ of geography variables (i.e. sea access, the presence of natural resources and the quality of the natural environment) do not show any statistically significant effect on the investors' plans to expand their businesses. Although the natural endowments of an area is a key locational factor of the Greek businesses (Rasvanis & Tselios, 2022), there is no evidence that these endowments also affect the business expansion in the Greek territory. In contrast, the ‘second-nature’ geography seems to have an impact on the investors' plans. More specifically, the proximity to similar firms (i.e. proximity to competitors) negatively affects the intention of entrepreneurs to expand their firms in the Greek territory, because OR < 1 and it is statistically significant. This is likely to indicate that firms which are in close proximity to other firms within the same or a similar sector have not benefited enough from the localisation externalities and therefore entrepreneurs do not intend to expand their businesses. A plausible explanation for this finding is that the benefits of local competition, which are maximised in the regions where specialised industries are concentrated, are based on imitation and innovation (Gustavsson, 2003). However, the empirical study of Filippopoulos and Fotopoulos (2022) shows that these characteristics are less profound in the lagging EU regions, such as in the Greek regions. The urbanisation economies proxied by the proximity to the capital do not affect the business expansion, because OR is not statistically significant. The location of Greece on the world map seems to be a critical factor for the expansion of businesses, because OR > 1 and it is statistically significant. We can infer that since Greece is located at the maritime crossroads of three continents, Greek businesses have a potential for future commercial development. The variables used to measure the effect of the quality of institutions (i.e. the control of corruption and the quality of local governance) have no statistically significant effect on the future planning of entrepreneurs about expanding. This is likely to denote that both domestic and foreign entrepreneurs are aware of the relatively low institutional quality of the country.

As for the controls, there is evidence that entrepreneurs with more than one establishment in the country would like to further expand their investment. The size of the business seems to play a role in investors' plans, as medium-sized businesses are more likely to be expanded than the very small, small and large ones. The number of establishments seems to be a very important parameter for the decision of the entrepreneurs to further expand their businesses in Greece. There is also strong evidence that businesses consisting of more than one establishment are more likely to further expand their activities. A possible explanation is that these businesses have greater financial strength and, at the same time, more experience in extending their facilities within Greece than other businesses.

### Business selling

Table 4 presents the regression results of the multinomial logistic model for the business selling.^6^ We report the McFadden’s pseudo-R^2^, which is expressed by the formula:${{R}^{2}}_{McFadden}=1-\frac{\mathrm{ln}({L}_{Model})}{\mathrm{ln}({L}_{0 })}$, where ${L}_{Model}$ is the value of the maximum likelihood of the applied model (with the predictor variables) and ${L}_{0}$ is the value of the maximum likelihood of a model without the predictor variables. This index is preferable to other indexes (e.g. the Cox and Snell pseudo-R^2^ or the Nagelkerke pseudo-R^2^), as $\mathrm{ln}({L}_{0 })$ better simulates the residuals in a linear regression than others. Nor did we find any evidence for multicollinearity, as the Variance Inflation Factor (VIF) values are lower than 1.5.^7^

**Table 4:** Multinomial logistic regression results for business selling

Do you intend to sell your business to an investor in the near future ?				
	Maybe	Yes, to a Greek investor	Yes, to a foreign investor	No (base category)
*‘First-nature’ geography*				
Sea access	0.993	2.026***	0.864	
Natural resources	1.012	1.096	0.926	
Natural environment	0.996	2.000**	0.972	
*‘Second-nature’ geography*				
Proximity to competitors	0.947	0.309***	0.960	
Proximity to the capital	1.022	0.368***	0.680***	
Country location	0.919*	1.378**	1.119	
*Institutions*				
Corruption	1.089	0.586	0.913	
Local governance	0.970	4.155*	1.021	
*Controls*				
Tax policy	1.134	0.709	1.289	
Infrastructure	1.093	2.446**	1.328	
Domestic	base	base	base	
Foreign	0.730	0.001***	2.809	
Manufacturing	base	base	base	
Services	1.416*	0.025*	1.307	
Very small firm	base	base	base	
Small firm	0.831	0.890	0.806	
Medium firm	0.679	41.856***	0.287	
Large firm	0.836	0.001***	0.457	
Number of Establishments = 1	base	base	base	
Number of Establishments > 1	1.093	0.356	1.224	
Constant	0.496	0.001**	0.027	
Observations			742	
Wald chi^2^			701.75	
Prob > chi^2^			0.0000	
Pseudo R^2^			0.0644	
Log likelihood			− 548.204	

^*^p < 0.10; **p < 0.05; ***p < 0.01

The regression results show strong evidence that both geography and institutions matter for the business selling to the Greek investors. The ‘first-nature’ of geography factors of access to the sea and the quality of the natural environment are important factors in reaching a sale agreement with a domestic investor. This might indicate that the domestic investors, who are well acquainted with an area, want to take advantage of these natural endowments and acquire a business that can be further developed based on these endowments. For example, a domestic investor may want to take advantage of the maritime access of the region where the company is located to carry out export activities or may want to gain access to raw materials which are abundant in this region for its production process.

Τhe ‘second-nature’ of geography factors show mixed results. It is the geographical location of Greece (OR > 1) that is the most likely to influence the sale agreement of the investment to a Greek investor, while the proximity to similar firms and the proximity to the Greek capital (OR < 1) are less likely to motivate the seller and buyer to enter into such an agreement. As for the role of institutions, the local government seems to be able to influence a business-selling decision, albeit at a 10% level of significance, but the existence of corruption does not seem to have any effect.

The only geographic factor that affects the decision of an entrepreneur to sell his/her business to a foreign investor is the proximity to the Greek capital (Athens). An investment located close to the capital is more likely to be sold to a foreign investor than to a domestic investor, possibly because the domestic investors have better knowledge of the conditions prevailing in the Greek market compared to the foreign investors, and because the domestic investors have diagnosed external diseconomies of scale in Athens due to the over-concentration of people and activities in the greater area of the capital.

Τhe control variables indicate that most entrepreneurs who are not sure whether or not they want to sell their businesses are from the service sector. The size of the business and the number of establishments do not appear to be capable of influencing the entrepreneurs' decision to sell or maintain his/her business. The existence of quality infrastructure—as was expected—has a positive effect on the prospect of selling the business to a Greek investor. Finally, medium-sized businesses are more likely to be sold to a domestic investor.

## Discussion

This paper studies the role of geography and institutions in the business plans of entrepreneurs in Greece. The present study shows that geography indeed matter for the expansion or sale of businesses, while institutions play a minor role.

Some studies (e.g. Lu et al., 2020) stress the key role of physical geography in the expansion of foreign investments in a region or country. However, this study makes a comparison between the domestic and foreign investments, and indicates that sea access and natural environment affect more the plans of the domestic investors than those of the foreign ones. Furthermore, although some scholars (e.g. Deller & Conroy, 2017) argue that the performance of businesses is sensitive to natural resources, this study finds no evidence that natural resources affect the survival and expansion of businesses in the manufacturing and services sectors.

In contrast to the study by Neffke et al., (2012), who argue that localisation externalities do not matter for manufacturing plants survival, this study finds that localisation externalities do not benefit manufacturing and service businesses’ expansion, and they are a deterrent to business selling. Urbanisation externalities do not influence entrepreneurs' decisions to expand their businesses, but they negatively affect a possible agreement of selling the business to an investor. An important finding that is absent from the literature concerning business survival and growth, which this study stresses, is that of the importance of the relative geographical location of the country, which affects both the expansion and the sale of a business.

As regards the institutional impact on investors' decisions, we found weak evidence on the local governance impact on entrepreneurs' intention to sell their businesses to domestic investors. We also found no statistically significant effect of Greek institutions on the entrepreneurs' plans to expand their businesses. This finding is in line with the study by Tsiapa (2022), who concludes that any improvements in transparency, by reducing corruption, have a weak influence on businesses' performance. This finding can also be explained by the fact that the efforts to reduce corruption have so far not yielded the expected results (Transparency International, 2021).

## Conclusion

### Theoretical implications

Ιn this paper, we have explored the future plans of domestic and foreign investors regarding the possibility of expansion or selling their businesses in the Greek territory. We focused on whether geography and institutions, which are important locational factors of businesses (Marks-Bielska et al., 2021; Rasvanis & Tselios, 2022), could also affect the entrepreneurs' strategic plans for expansion or the sale of their businesses. To achieve this aim, we used primary data derived from a survey questionnaire sent to a range of managers/owners of the key sectors of the Greek economy.

This research shows that the localisation economies are inhibiting factors for business expansion, while the location of Greece is a significant incentive for expansion within the territory. The results also indicate that an investor who operates in Greece thinks about selling his/her business when everything in the Greek economy is ideal in terms of the sea access, the natural environment, the location of Greece and the local governance. Since the Greek investors have a better knowledge of the Greek economy than the foreign ones, there is evidence that the geographical and institutional factors above determine the business sales to the Greek investors only. Both the localisation and the urbanisation economies are disincentives for selling to a Greek investor.

### Limitations, policy and managerial implications

This study is not without limitations. The first limitation has to do with the data collection. The data were collected mainly before the Covid-19 pandemic and only a few at the onset of the pandemic. Thus, we do not know whether the pandemic changed the intention of entrepreneurs to expand or sell their businesses. The second one has to do with the location of business expansion. More specifically, we do not know where (i.e. in which areas) investors intend to expand their business.

Putting aside these limitations, this study offers findings that had not been emphatically highlighted in the previous literature which could be important guidelines for policy makers and managers. This study shows that the main factor that determines the future planning of businesses operating in Greece is the 'second-nature' geography, i.e. agglomeration economies and the location of the country in the global market. This suggests that managers could exploit the benefits of agglomeration economies for businesses—because these economies seem to discourage the business selling—and policies going forward could further exploit the importance of the country's geographical location on the world map to develop the existing investments and to attract new investors. Nevertheless, entrepreneurs and managers in Greece need to focus on the importance of its natural endowments (‘first-nature’ geography), and especially those of sea access and the natural environment, as they matter not only for business location (Rasvanis & Tselios, 2022) but also for business selling to domestic investors. The finding that local governance may attract the interest of the Greek investors in acquiring a business points to the key role of decentralisation in entrepreneurship (Rangus & Slavec, 2017). Therefore, the policies pursued in Greece could reinforce the role of local governance and give it a greater degree of autonomy and more financial tools. Finally, the very important role played by infrastructure in the intention of an entrepreneur to sell his/her business to a Greek investor, who is well aware of the development of infrastructure in the country, confirms the importance of the infrastructure and its improvement in stimulating business activities (Ngoma et al., 2021; Shome, 2013). Hence, managers and/or entrepreneurs need to exert pressure on decision-makers towards further improvement of telecommunications, but mainly transport infrastructure.

### Ideas for future research

Considerable light can be shed on the two limitations of this study by further analysis. First, since this survey was mainly conducted in the pre-pandemic period, its results could also be verified in the post-pandemic period. Second, since we do not know where investors intend to expand their business, future research on the location of expansion would be interesting. Other issues for future research include the impact of corporate tax system and its complexity on corruption, which is an important aspect of the quality of institutions, and how this affects the business plans. This is an interesting topic for future research for the Greek economy, because the possible interaction between taxes and corruption, as shown by previous studies (Alm et al., 2016; Liu & Feng, 2015), may affect the Greek entrepreneurial behaviour.

## Supplementary Information

**Additional file 1.** Appendices.

## Data Availability

Upon authors' request.

## Footnotes

1 As the geographical location of a country is a factor that represents an unvarying geographical feature, it could also be considered as a ‘first-nature’ of geography factor. However, we are more concerned with the country’s position in relation to neighbouring countries, to international markets or to regions with agglomeration economies. Therefore, in this study, we examine Greece’s geographical location as a ‘second-nature’ of geography factor.

2 The ability to check the time taken to complete a questionnaire was provided by the specialised application.

3 For the structure of the questionnaire and the questions used in the analysis, see Additional file 1: Appendix S1.

4 We removed respondents who answered 'I don't know/Ι don't answer'.

5 The beta coefficients are presented in Additional file 1: Appendix S2.

6 The beta coefficients are presented in Additional file 1: Appendix S3.

7 See Additional file 1: Appendix S4.
