# Supplementary material for: Do geography and institutions affect entrepreneurs’ future business plans? Insights from Greece
Source: J Innov Entrep. 2023 Feb 24;12(1):3. doi: 10.1186/s13731-023-00266-3 (PMC9951831; doi:10.1186/s13731-023-00266-3)
Supplement: Supplementary file 1 — Additional file 1. Appendices. [file 13731_2023_266_MOESM1_ESM.docx]

**Appendix 1: Survey questions used in the analysis**

| ***Profile of the Investment*** |
| --- |
| **1.** Which economic sector does your company belong to? |
| a) Manufacturing-Industry (Secondary) b) Services (Tertiary) c) Energy |
| **2.** Which subsector of the secondary sector does your company belong to? ­­________ (***only for companies of the secondary sector***) |
| **3.** Which subsector of the tertiary sector does your company belong to? |
| a) Tourism b) Transport c) Other (Please specify) |
| **4.** Investment size (according to regulation 2003/361 / EC): |
| a) Very small (up to 10 employees) |
| b) Small (10-49 employees) |
| c) Medium (50-249 employees) |
| d) Large (250+ employees) |
| **5.** Prefecture (NUTS 3 region) in which your company is located: __________ |
| **6.** Indicate the type of installation: |
| a) Headquarters of the company, production unit and sales in the same location |
| b) Headquarters of the company in one location AND production unit and sales in another location |
| c) Headquarters of the company and production unit in one location AND sales in a different location |
| d) Headquarters of the company and sales in the same location AND production unit in another location |
| **7.** Number of establishments that make up your business: |
| a) One b) More than one |
| **8.** Does your business have only Greek investors? |
| * Yes, it has only Greek investors ** No, there are foreign investors  **9.** State the percentage (%) of foreign ownership (with voting rights) in the company's capital share **(*only for FDI*)**:  a) Less than 10% b) 10%-50% c) More than 50% |
| ***Intention to expand/sale*** |
| **10.** Do you intend to further expand your existing business activity in the Greek territory?  a)Yes  b) No  c) I don’t know/ I don’t answer  **11.** Do you intend to sell your business to an investor in the near future?  a) Maybe  b) Yes, to a Greek investor  c) Yes, to a foreign investor  d) No |
| ***The role of geography in the location of the investment*** |
| Express your opinion on the following statements: |
| **12**. Direct access to the sea and the existence of harbor were very important factors for the location of the investment. (1-7 Likert scale, 1= Strongly disagree, 7 = Strongly agree) |
| **13.** The existence of natural resources (e.g. soil and subsoil) has been a critical factor in selecting the location of the investment. (1-7 Likert scale, 1= Strongly disagree, 7 = Strongly agree) |
| **14.** How important was the quality of the natural environment to the installation of your investment? (1-7 Likert scale, 1= Not important at all, 7= Very important) |
| **15.** Proximity of your company to similar/competitive companies has influenced the location choice of the investment. (1-7 Likert scale, 1= Strongly disagree, 7 = Strongly agree) |
| **16.** Proximity to the capital of Greece (Athens) or more broadly to the region of Attica affected the location choice of the investment. (1-7 Likert scale, 1= Strongly disagree, 7 = Strongly agree) |
| **17.** Greece’s location on the world map (i.e. Greece's neighborhood with the rest of the Balkan countries and the fact that it is a crossroads of three continents) was a determining factor in location choice of the investment. (1-7 Likert scale, 1= Strongly disagree, 7 = Strongly agree) |
| ***Evaluation of other determinants*** |
| **18.** State your opinion on the influence of these factors in the establishment of an investment in Greece: |

|  | Not important | | |  | Very important | | |
| --- | --- | --- | --- | --- | --- | --- | --- |
| Factor | 1 | 2 | 3 | 4 | 5 | 6 | 7 |
| Corruption |  |  |  |  |  |  |  |
| Local governance |  |  |  |  |  |  |  |
| Tax policy |  |  |  |  |  |  |  |
| Quality of infrastructure |  |  |  |  |  |  |  |

**Appendix 2:** **Beta coefficients for business expansion**

| **Do you intend to further expand your existing business activity in the Greek territory?** | | |
| --- | --- | --- |
|  | **Yes** | **No (base category)** |
| **‘First-nature’ geography** |  |  |
| Sea access | 0.063 |  |
| Natural resources | -0.076 |  |
| Natural environment | -0.026 |  |
| **‘Second-nature’ geography** |  |  |
| Proximity to competitors | -0.144*** |  |
| Proximity to the capital | 0.083 |  |
| Country location | 0.141*** |  |
| **Institutions** |  |  |
| Corruption | 0.150 |  |
| Local governance | -0.025 |  |
| **Controls** |  |  |
| Tax policy | -0.038 |  |
| Infrastructure | -.0134* |  |
| Domestic | base |  |
| Foreign | -0.038 |  |
| Manufacturing | base |  |
| Services | -0.173 |  |
| *Very small firm* | base |  |
| *Small firm* | -0.096 |  |
| *Medium firm* | 0.727** |  |
| *Large firm* | 0.744 |  |
| Number of Establishments =1 | base |  |
| Number of Establishments >1 | 0.932*** |  |
| Constant | 0.432 |  |
| Observations | 557 |  |
| Prob>chi^2^ | 0.0000 |  |
| Pseudo R^2^ | 0.0899 |  |
| Log likelihood | -348.298 |  |

^*^p < 0.10; **p < 0.05; ***p < 0.01

**Appendix 3: Beta coefficients for business selling**

| **Do you intend to sell your business to an investor in the near future?** | | | | |
| --- | --- | --- | --- | --- |
|  | **Maybe** | **Yes, to a Greek investor** | **Yes, to a foreign investor** | **No (base category)** |
| **‘First-nature’ geography** |  |  |  |  |
| Sea access | -0.007 | 0.563*** | -0.161 |  |
| Natural resources | 0.018 | 0.025 | -0.039 |  |
| Natural environment | -0.005 | 0.551** | -0.024 |  |
| **‘Second-nature’ geography** |  |  |  |  |
| Proximity to competitors | -0.045 | -0.960*** | -0.025 |  |
| Proximity to the capital | 0.020 | -0.895*** | -0.383*** |  |
| Country location | -0.055* | 0.314** | 0.113 |  |
| **Institutions** |  |  |  |  |
| Corruption | 0.086 | -0.452 | -0.038 |  |
| Local governance | -0.027 | 1.367* | 0.028 |  |
| **Controls** |  |  |  |  |
| Tax policy | 0.125 | -0.534 | 0.258 |  |
| Infrastructure | 0.084 | 1.068** | 0.259 |  |
| Domestic | base | base | base |  |
| Foreign | -0.305 | -12,589*** | 1.063 |  |
| Manufacturing | base | base | base |  |
| Services | 0.334* | -2.439* | 0.604 |  |
| Very small firm | base | base | base |  |
| Small firm | -0.200 | 0.437 | -0.271 |  |
| Medium firm | -0.406 | 4.121*** | -1.273 |  |
| Large firm | -0.215 | -9.325*** | -0.795 |  |
| Number of Establishments =1 | base | base | base |  |
| Number of Establishments >1 | 0.111 | -0.983 | 0.209 |  |
| Constant | -0.641 | -15.296** | -3.572 |  |
| Observations | 742 | | | |
| Prob>chi^2^ | 0.0000 | | | |
| Pseudo R^2^ | 0.0644 | | | |
| Log likelihood | -548.204 | | | |

^*^p < 0.10; **p < 0.05; ***p < 0.01

**Appendix 4: Variance inflation factor (VIF) values**

| ***Dependent variable: Sea access*** | |
| --- | --- |
| **Independent variables** | **VIF** |
| Country location | 1.38 |
| Natural resources | 1.30 |
| Local governance | 1.28 |
| Infrastructure | 1.27 |
| Proximity to competitors | 1.27 |
| Proximity to the capital | 1.27 |
| Tax policy | 1.22 |
| Business Size | 1.22 |
| Natural environment | 1.21 |
| Corruption | 1.17 |
| Number of establishments | 1.16 |
| Origin (Domestic or foreign) | 1.10 |
| Sector | 1.08 |
| **Mean VIF** | **1.22** |
